# Inpatient and Emergency Room Ophthalmology Consultations at a Tertiary Care Center

**DOI:** 10.1155/2019/7807391

**Published:** 2019-02-14

**Authors:** Daniel J. Oh, Levi N. Kanu, Judy L. Chen, Ahmad A. Aref, William F. Mieler, Peter W. MacIntosh

**Affiliations:** University of Illinois at Chicago, Department of Ophthalmology and Visual Sciences, Chicago, IL, USA

## Abstract

**Background:**

An ophthalmology consultation service is of significant benefit to patients in the hospital and is an instructive component of a residency education program. Ophthalmology consultations in a hospital present unique challenges to those seen in an outpatient clinic, for which the consulting ophthalmologist should be prepared. The purpose of this study was to profile the emergency room and inpatient ophthalmology consultations seen at an academic institution.

**Methods:**

A prospective study of 581 patients was conducted on inpatient and emergency room ophthalmology consultations at the University of Illinois at Chicago over twelve months. Characteristics such as the consulting service, type of and reason for consultation, subspecialty staffing service, diagnosis, and suitability for in-hospital evaluation were recorded.

**Results:**

Consultations were received from either inpatient wards (59.4%) or the Emergency Department (40.6%). The most common inpatient consulting services were internal medicine (22%), followed by neurosurgery (16%) and neurology (7%). All the consultations were categorized as acute (72.3%), chronic (6.0%), or screening (21.7%). Consultations categorized as screening included papilledema (31.0%), fungemia (20.6%), syndromic evaluation (19.8%), visual field evaluation (17.5%), and miscellaneous evaluation (11.1%). We classified the ophthalmic diagnoses into 63 unique diagnoses. Amongst the ophthalmic subspecialties, neuro-ophthalmologic diagnoses were the most common (32.0%), followed by retina (20.1%) and cornea (19.4%). Neuro-ophthalmology had the highest proportion of screening consultations (36.6%), while glaucoma had the least overall number of consultations (10.1%), and the least proportion of screening consultations (3.6%). A significant proportion of nonacute consultations (19.0%) was deemed to be more suitable for outpatient evaluation.

**Discussion:**

Consultation databases can be useful in preparing trainees for in-hospital clinical care. A wide range of ocular pathologies may present to the ophthalmology consultant, from acute trauma to screening for systemic syndromes. Some consultations may be more suitable for outpatient evaluation which may help optimize patient care.

## 1. Introduction

Inpatient consultation services are an important part of residency education and a critical component of providing clinical care for patients. Ophthalmic issues that arise in-hospital present unique circumstances for the evaluating ophthalmologist. A bedside ophthalmic examination may require the use of portable instruments which may be limited in capabilities or less technically precise than dedicated outpatient equipment. Equipment or supplies may not be readily available. Patients may also be delirious, uncooperative, or unresponsive, making the ophthalmic examination difficult. On a busy service, the consultant may need to abridge one patient's examination in order to focus on a more acute one. Previous studies have profiled inpatient ophthalmology consultations at various institutions. The types of consultations vary greatly based on the institution; some programs primarily see chronic eye problems on consultation [1, 2], whereas consultations at Level 1 trauma centers are skewed towards trauma [3, 4]. A familiarity with the various levels of patient acuity will aid in the efficiency of the ophthalmology consultant.

Prior descriptive studies have improved our understanding of inpatient ophthalmology consultation services. One study recommended that residency training should focus on skills such as using a portable slit lamp and indirect ophthalmoscope [5]. Another study suggested that better triaging of patients could be achieved if the consulting service checked the visual acuity [2]. For the sake of efficiency, some consultations may be deferred to the outpatient setting [1, 5]. This practice allows for a reallocation of resources based on the presumed urgency of the consultation and may increase the overall efficiency of a consultation service. However, the determination of which consultations are safe to be deferred may prove to be difficult. Further analysis and characterization of consultations may shed new light into improving efficiency of the consultation services.

In this study, we perform a comprehensive analysis of the ophthalmology consultations seen at our institution. We characterize the wide variety of ophthalmology consultations encountered in our hospital and investigate whether some consultation requests may be safe and more suitable for a scheduled outpatient evaluation. Ultimately, we seek to provide further insight into the optimization of consultation services. We believe this will lead to an overall improved in-hospital experience for patients and providers.

## 2. Methods

This was a prospective, observational study of ophthalmology consultations at the University of Illinois Hospital over the period of November 1, 2017, to October 31, 2018. The study received institutional review board approval. No identifiable patient information was collected. This research followed the tenets of the Declaration of Helsinki.

Consultations were requested by various inpatient and emergency medical teams. Consultations were received electronically or via paging system. As a hospital policy, all consultations requesting ophthalmology evaluation were accepted and recorded by the resident on the consultation rotation. Residents rotating on the consultation service were instructed to record patient demographics, consulting service and location, and reason for consultation, as part of the rotation guidelines. A designated consulting resident handled all new daytime consultations and was specifically trained on prospective data collection, whereas overnight and weekend consultations were handled by rotating residents. To aid in data consistency, only daytime consultation data were included in the study.

The emergency room was equipped with an ophthalmology examination room, complete with slit lamp, B-scan ultrasound, and applanation tonometer. Visual field testing and imaging such as fundus photography, OCT, and ultrasound biomicroscopy were available upon request in the outpatient clinic. Inpatient consultations were primarily evaluated with portable devices including portable slit lamp and electronic applanation tonometer but were brought to the emergency room for standard slit lamp examination as needed.

The consulting resident performed a complete ophthalmologic examination on each patient. Based on presumptive diagnosis, the resident would then determine the most appropriate subspecialty for staffing. A management plan was then devised with the staffing subspecialist and communicated with the consulting service.

Consultations were categorized into acute, chronic, and screening types. Acute was defined as new or worsened ocular symptoms. Chronic was defined as known prior ocular diagnoses without any change in symptoms during admission. Screening consultations were defined as systemic conditions prompting ophthalmic examination without ocular symptoms.

Based on the reason for consultation, each consultation was assessed for suitability for in-hospital rather than outpatient evaluation. Consultations deemed less suitable for in-hospital evaluation, herein referred to as “*non-essential*” inpatient consultations, met the following criteria: (1) nonacute, (2) inpatient management would not be changed based on findings, and (3) inpatient evaluation would be less complete than outpatient evaluation.

## 3. Results

**Figure 1:**
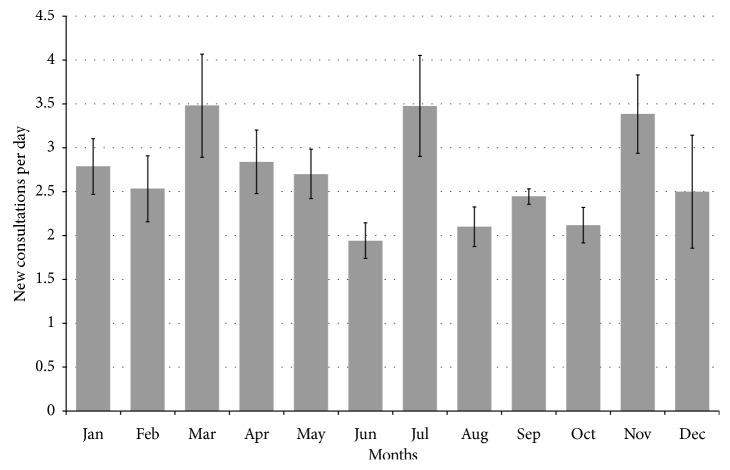
New consultations/day by month (mean ± standard deviation).

A total of 581 individual ophthalmology consultations were received over the study period. 52.3% were female and 47.6% were male. Patients' age ranged from 0 (newborn) to 87. There was no statistically significant seasonal variation with regard to number of daily consults (range 2.3 to 3.6 daily consults, *p*=0.34); however, monthly variation was statistically significant (range 1.9 to 3.5 daily consults, *p*=0.046) (Figure 1).

**Figure 2:**
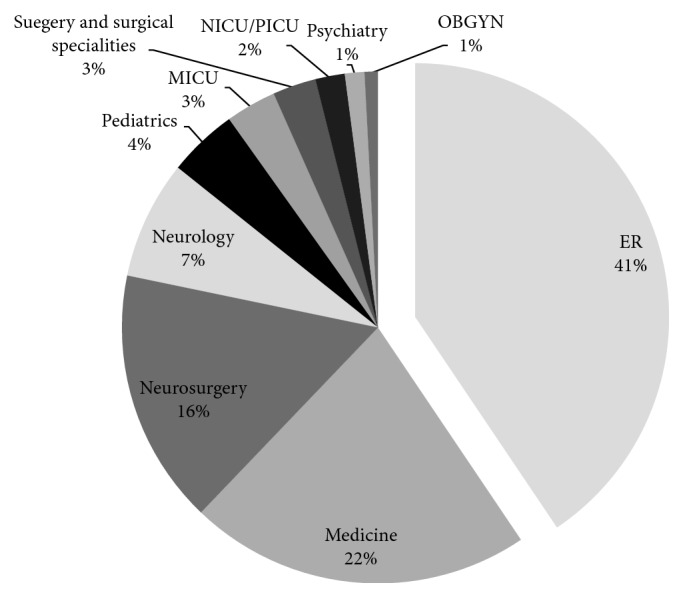
Distribution of consulting services. Ophthalmology consultations were requested from inpatient or emergency room services. ER = Emergency Room; MICU = medical intensive care unit; NICU = neonatal intensive care unit; PICU = pediatric intensive care unit; OBGYN = obstetrics and gynecology.

Consultations were placed from either the inpatient wards (59.4%) or the Emergency Department (40.6%). The most common inpatient consulting services were internal medicine (21.5%), followed by neurosurgery (16.2%) and neurology (7.4%) (Figure 2). However, consultation requests spanned the breadth of clinical subspecialties including pediatrics, medical intensive care unit (MICU), general surgery and surgical subspecialties (orthopaedic surgery, otolaryngology, urology, and oral and maxillofacial surgery), neonatal and pediatric intensive care units (NICU and PICU), psychiatry, and obstetrics and gynecology (OB/GYN).

Reasons for consultation were divided into 10 categories, most commonly, vision change (30.3%), eye pain (19.8%), and periorbital pain and swelling (12.0%). Other categories included trauma (10.0%), papilledema (8.4%), tumor (4.8%), fungemia (4.8%), syndromic evaluation (4.0%), optic neuritis (1.4%), and others (4.5%) (Table 1).

Consultations were categorized as acute (72.3%), chronic (6.0%), or screening (21.7%) (Table 1). Screening consultations were further subdivided into the following categories: papilledema (31.0%), fungemia (20.6%), syndromic evaluation (19.8%), visual field evaluation (17.5%), and miscellaneous evaluation (11.1%). Positive ocular findings were observed in 0% of fungemia consultations, 20.5% of papilledema consultations, 52.0% of syndromic consultations, 27.3% of visual field consultations, and 14.3% of miscellaneous consultations. For acute and chronic consultations, the most common reasons for consultation included vision change (30.3%), eye pain (19.8%), and periorbital pain and swelling (12.0%) (Table 1).

A primary ocular diagnosis was found in 431 patients (74.2%), comprising 63 unique diagnoses (Table 2). 105 patients (18.0%) had a primary corneal diagnosis, 100 patients (17.2%) had a neuro-ophthalmologic diagnosis, 95 patients (16.3%) had an orbit/oculoplastics diagnosis, 75 patients (12.9%) had a retinal diagnosis, 25 patients (4.3%) had a glaucoma diagnosis, and 31 patients (5.3%) had a diagnosis that could not be categorized into a specific subspecialty. 150 patients (25.8%) were ultimately found not to have any ocular pathology; that is, patients with visual or ocular symptoms ultimately were found not to have ocular pathology, or asymptomatic patients with negative screening examinations.

**Figure 3:**
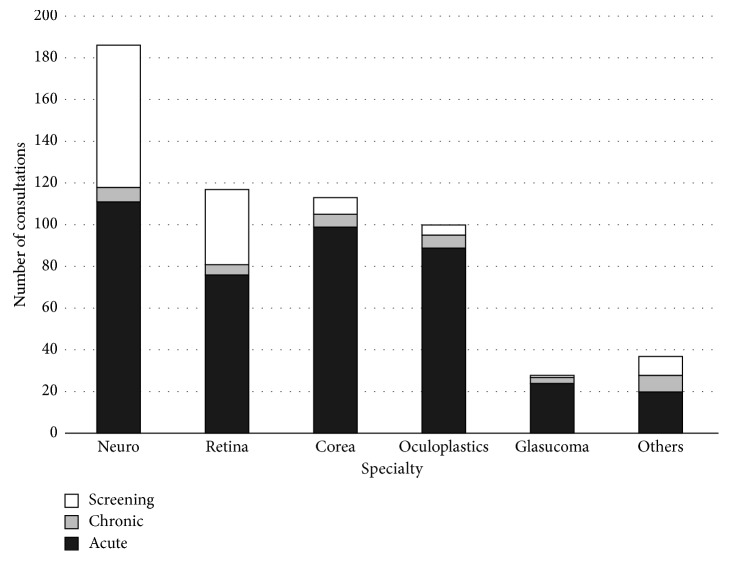
Diagnosis by subspecialty and consultation type within each subspecialty.

All patients were expected to be staffed by a subspecialist attending physician or residency-trained fellow. The most commonly requested subspecialists were neuro-ophthalmologist (32.0%), retina specialist (20.1%), and cornea specialist (19.4%) (Figure 3). Five hundred and fifty-seven patients (96.0%) were staffed by one subspecialist, 20 patients (3.4%) were staffed by two subspecialists, and 4 patients (0.6%) were staffed by three subspecialists.

Some reasons for consult resulted in a diverse range of possible diagnoses. For example, patients complaining of vision change were ultimately diagnosed with neuro-ophthalmology (47.8%), retina (29.7%), cornea (11.0%), glaucoma (2.7%), and oculoplastics (1.6%) pathologies. Similarly, patients with eye pain had cornea (60.9%), glaucoma (17.4%), retina (10.4%), neuro-ophthalmology (5.2%), and oculoplastics (3.5%) diagnoses.

A substantial portion of the nonacute consultations (*n*=44, 19.0%) was deemed to be more suitable for outpatient evaluation or “nonessential” inpatient consultations. All but two nonessential consultations (95.4%) came from inpatient services. The majority of nonessential consultations (84.0%) were screening examinations, of which the majority were for confrontational visual field (CVF) testing (56.8%) or for pediatric examinations (27.6%). The most common reasons for nonessential CVF testing included requests for baseline preoperative examinations on patients admitted for surgical resection of known sellar masses who had been diagnosed in the outpatient setting. Nonessential pediatric examinations comprised asymptomatic children with syndromes associated with ocular findings, but without an apparent ocular abnormality prompting consultation.

## 4. Discussion

Ophthalmology is largely an outpatient specialty, and much of our equipment and testing are designed for maximum efficiency in that setting. However, a variety of ophthalmic pathology may be present in an emergent setting, or in patients who are hospitalized for nonophthalmic reasons which may present special challenges for obtaining an optimum evaluation of these patients.

Our study demonstrates the substantial number of inpatient and emergency consultations that may be encountered by the consulting ophthalmologist. These consultations include a wide variety of ophthalmic and systemic pathologies requiring multidisciplinary management and multi-subspecialty care. Consulting ophthalmologists should tailor their training to the evaluation and treatment of the most common pathologies encountered by our service, including dry eye syndrome, corneal abrasion, and optic neuritis (Table 2). Similarly, our results will guide the ophthalmologist to be prepared for the co-management of patients with the most frequently consulting services.

The rate of consultations seen by our service was characterized by statistically significant monthly variation, with peaks in the months of March, July, and November and troughs in June, August, and October (Figure 1, *p*=0.046). It is not immediately apparent why such variation exists, but it is likely multifactorial. For instance, the single busiest day by consultations was July 4, during which 13 new ophthalmology consultations were requested, and most of these consultations were for firework-related ocular trauma. However, there was no significant variation based on season, and there was no trend over chronological time (Figure 1).

Most of our consultations were requested by inpatient services, and as with prior studies, medicine, neurology, and neurosurgery accounted for the majority of our inpatient consultations. Nonetheless, consultations were requested from a wide range of specialties, underscoring the important role of ophthalmology in inpatient medicine, despite being a primarily outpatient specialty. The inpatient consultant should be prepared to discuss diagnosis, treatment, and relevant specialty-specific implications with all possible consulting specialties.

When consultations were categorized by relevant ophthalmic subspecialty, neuro-ophthalmology was the most frequently consulted subspecialty. However, almost all of these differences were attributed to screening examinations. Similarly, while the retina subspecialty had the second highest number of consultations, it would only be fourth highest when excluding screening examinations. In contrast, there were relatively few glaucoma consultations and almost no screening glaucoma examinations. It is unclear how this distribution compares to that in other institutions, as subspecialty distribution has not been reported in prior studies.

Screening examinations accounted for a significant number of consultations across all ophthalmic subspecialties and warrants further discussion. Papilledema screening is a common consultation request for the inpatient ophthalmology service [1, 4]. Many such examinations involved assessing a patient complaining of headache for the presence of papilledema to assist in the evaluation for elevated intracranial pressure. Fundus examination findings must be interpreted with an understanding of clinical context. Patients may have elevated intracranial pressure without apparent papilledema [6, 7]. Therefore, while the finding of papilledema in a patient with headache is highly suggestive of elevated intracranial pressure, the lack of papilledema does not rule it out. Importantly, vision loss in early papilledema may be subtle, and the lack of visual complaints does not rule out the possibility of papilledema. Ultimately, the evaluation for papilledema is requested in order to determine the need for additional imaging or lumbar puncture, but some of the subtle visual changes are only measurable with formal automated visual field testing, which is not easily available in the inpatient setting. While papilledema screening may continue to be a common and important screening examination, it is important for the ophthalmologist to understand these limitations during their examination and discussion with the consulting service.

Another common neuro-ophthalmology screening examination involved performing confrontational visual fields to assess for a visual field deficit. This was typically done in the setting of a known pituitary mass, in which pre- and postmass resection vision testing was requested. However, confrontational visual field (CVF) testing has a low sensitivity, especially when performed individually, and the utility of this as a screening tool is questionable [8–10]. CVF testing may vary widely between examiners and does not allow for reliable longitudinal monitoring of visual field deficits. Furthermore, it is unclear whether CVF testing by an ophthalmology consultant would necessarily be more accurate than examination by the consulting service. In patients undergoing scheduled surgery for known lesions along the visual pathway, CVF is likely an inappropriate screening examination. Formal automated perimetry should be scheduled before and after surgical interventions, and ophthalmology consultation for CVF testing may only be appropriate in cases of acute or dramatic changes in vision, or in which the pretest probability of a CVF deficit is high.

The retina subspecialty also encountered a significant amount of screening examinations, specifically for fundus examination of patients with fungemia. Consistent with prior studies, fungemia is a common reason for inpatient consultation. However, prior studies have called into question the practice of screening examinations on all fungemic patients [11]. The rate of positive findings found in patients consulted for rule-out fungal endophthalmitis is variable, ranging from 0 to 7.7% in the literature [4, 11, 12], and our data fall within this range.

Our results indicate that a significant number of consultations may be more appropriately deferred to the outpatient setting. *Nonessential* consultations included a substantial number of CVF screening tests and pediatric examinations. This determination was based on reason for consultation and did not depend on the examination findings or diagnosis. Therefore, a list of generally *nonessential* consultations may be useful to share with consulting teams and to aid in triaging and resource allocation. However, it is important to realize that while our definition of *nonessential* was purposefully objective, the determination may not account for more social and logistical aspects of patient care. For example, a patient with chronic glaucoma, without visual complaints, may merit inpatient consultation if they have been lost to follow-up and are unlikely to show to an outpatient ophthalmology appointment, despite the inability to perform adequate screening while inpatient. Similarly, a consultation may indeed be essential in the setting of transportation issues, lack of local access to healthcare, and financial issues affecting follow-up.

Inpatient ophthalmology consultation services vary by program, but there are some generalizable concepts for which residency education should be tailored. One study suggested that inpatient consultation training should consist of four training areas: (1) examination techniques; (2) ophthalmic manifestations of common inpatient diseases; (3) ophthalmic testing, treatment, and procedures; and (4) ocular infections [5]. The inpatient ophthalmology consultant should be prepared for a wide range of possible pathologies; a patient presenting with “eye pain” could end up with a primary ocular diagnosis from any ophthalmology subspecialty. Our results also suggest that a focus on screening examination training may be of particular importance. Correctly identifying the “rule-out” diagnoses (such as papilledema and fungal endophthalmitis) and counselling the consulting team properly on the significance of positive or negative findings are important. For example, consultation reports should explicitly state that the absence of papilledema does not rule out the possibility of intracranial hypertension. Furthermore, discussion with the primary team regarding the poor reliability of confrontational visual fields and appropriate reasons for consultation may lead to more resource-sensitive consultation screening practices.

Our study did not include overnight and weekend consultations, although these time periods likely contribute significantly to overall number of consultations, and the study of this would be useful in determining the importance of having ophthalmology consultation services available at all hours. Furthermore, overnight and weekend consultations may differ in patient population and pathology, and comparison with daytime consultations would be useful. While our study identified *nonessential* consultations, a study including the evaluation of consultations resulting in changed management would be instructive.

## 5. Conclusion

In this study, we have characterized inpatient and emergency room ophthalmology consultations seen at our institution. While ophthalmology consultations likely vary significantly by hospital, a knowledge of the types of consultations and their relative distributions may help in the structuring of residency consultation programs.

## Figures and Tables

**Table 1:** Consultation type by acute, chronic, and screening (top) and reason for consults for screening consultations (bottom).

*Type of consult*	
Acute	420 (72%)
Chronic	35 (6%)
Screening	126 (22%)
Screening type	Positive findings
Fungemia	0/26 (0%)
Papilledema	8/39 (21%)
Syndromic evaluation	13/25 (52%)
Visual field	6/22 (27%)

*Reason for consult*	
Vision change	182 (31.3%)
Eye pain	115 (19.8%)
Periorbital pain and swelling	70 (12.0%)
Trauma	58 (10.0%)
Papilledema	49 (8.4%)
Fungemia	28 (4.8%)
Tumor	28 (4.8%)
Syndromic evaluation	23 (4.0%)
Others	28 (4.8%)

**Table 2:** Primary ophthalmological diagnoses, categorized by subspecialty.

*Cornea and external disease (n*=105)	
30	Dry eye syndrome
23	Corneal abrasion
13	Subconjunctival hemorrhage
12	Conjunctivitis
10	Corneal ulcer
6	Hyphema
12	Others

*Neuro-ophthalmology (n*=100)	
20	Optic neuritis
18	Cranial nerve palsy
18	Optic neuropathy
18	Papilledema screening
11	Visual field defect screening
10	Cerebrovascular accident
5	Others

*Oculoplastics/orbit (n*=95)	
14	Orbital wall fracture
12	Preseptal cellulitis
11	Orbital cellulitis
8	Orbital mass
7	Thyroid eye disease
6	Chalazion
6	Lid laceration
29	Others

*Retina and uveitis (n*=75)	
16	Vitreous hemorrhage
13	Uveitis
10	Retinal hemorrhage
9	Retinal detachment
6	Central retinal artery occlusion
6	Traumatic iritis
15	Others

*Glaucoma (n*=25)	
12	Angle closure glaucoma
6	Neovascular glaucoma
3	Primary open angle glaucoma
3	Secondary postoperative glaucoma
1	Blebitis

*Others (n*=31)	
12	Open globe injury
11	Syndromic evaluation
8	Cataract

*No ocular pathology (n*=150)	
150	No ocular pathology

## Data Availability

The summarized data used to support the findings of this study are included within the article. Raw data are available from the corresponding author upon request.
